# Brillouin-Scattering Induced Noise in DAS: A Case Study

**DOI:** 10.3390/s23125402

**Published:** 2023-06-07

**Authors:** Boris G. Gorshkov, Denis E. Simikin, Alexey E. Alekseev, Mikhail A. Taranov, Konstantin M. Zhukov, Vladimir T. Potapov

**Affiliations:** 1 Prokhorov General Physics Institute of the Russian Academy of Sciences, Vavilov Street 38, 119991 Moscow, Russia; 2 Petrofiber, LLC, Klinsky Proezd 7, 301664 Novomoskovsk, Russia; 3 Kotelnikov Institute of Radio-Engineering and Electronics, Fryazino Branch, Russian Academy of Sciences, Vvedenskogo Square 1, 141190 Fryazino, Russia

**Keywords:** phase-sensitive optical time-domain reflectometer (phase-OTDR), fiber optic sensors, distributed acoustic sensing (DAS), spontaneous Brillouin scattering (SpBS), stimulated Brillouin scattering (SBS)

## Abstract

In the paper, the effect of spontaneous Brillouin scattering (SpBS) is analyzed as a noise source in distributed acoustic sensors (DAS). The intensity of the SpBS wave fluctuates over time, and these fluctuations increase the noise power in DAS. Based on experimental data, the probability density function (PDF) of the spectrally selected SpBS Stokes wave intensity is negative exponential, which corresponds to the known theoretical conception. Based on this statement, an estimation of the average noise power induced by the SpBS wave is given. This noise power equals the square of the average power of the SpBS Stokes wave, which in turn is approximately 18 dB lower than the Rayleigh backscattering power. The noise composition in DAS is determined for two configurations, the first for the initial backscattering spectrum and the second for the spectrum in which the SpBS Stokes and anti-Stokes waves are rejected. It is established that in the analyzed particular case, the SpBS noise power is dominant and exceeds the powers of the thermal, shot, and phase noises in DAS. Accordingly, by rejecting the SpBS waves at the photodetector input, it is possible to reduce the noise power in DAS. In our case, this rejection is carried out by an asymmetric Mach-Zehnder interferometer (MZI). The rejection of the SpBS wave is most relevant for broadband photodetectors, which are associated with the use of short probing pulses to achieve short gauge lengths in DAS.

## 1. Introduction

Phase-sensitive optical time domain reflectometer (phase-OTDR) is a promising and modern method for the simultaneous detection of spatially distributed dynamic events. The phase-OTDR sensing element is usually a widely available single-mode optical fiber. Fiber optic cable is placed in an elastic medium along an extended object and perceives vibrations with high sensitivity provided by the interferometric measurement principle. For this reason, phase-OTDR is commonly referred to as a distributed vibration sensor (DVS) or a distributed acoustic sensor (DAS). In the last decade, DAS has been widely used in various fields, from perimeter or pipeline security [1,2] and traffic monitoring [3] to seismic surveys [4,5,6] and seismology [7,8,9,10,11,12]. The increasing popularity of DAS is facilitated by its definite advantages in comparison with traditional seismic electrical systems, such as ease of deployment, large quantities of data obtained at relatively low costs, the possibility of using existing fiber optic infrastructure, and economic efficiency. At the same time, DAS is noticeably less sensitive and has a smaller dynamic range compared to conventional sensors.

The DAS is usually characterized by strain sensitivity (or strain resolution), which determines the minimum possible detectable strain of a fiber under test (FUT) in the presence of noise [11,13,14]. A convenient unit for measuring the DAS strain sensitivity is the strain amplitude spectral density (ASD) of noise described in $\varepsilon /\sqrt{Hz}$ (where ε is a dimensionless value that denotes the longitudinal strain). Strain sensitivity shows the minimum possible detectable strain amplitude within a certain frequency band and at a given DAS pulse repetition rate. Sensitivity improvement is one of the important directions of DAS technology development. In [11], the use of active laser frequency stabilization and compensation of the time delay between interfering backscattered fields made it possible to obtain a strain sensitivity of 40 $p\varepsilon /\sqrt{Hz}$ at 1 Hz with a 2 kHz pulse repetition rate and the DAS gauge length 18 m. The wavelength scanning approach proposed in [13] demonstrated a sensitivity of 100 $p\varepsilon /\sqrt{Hz}$ at 10 Hz with a 2 kHz pulse repetition rate and 5 m spatial resolution. The DAS with weighted averaging of responses over different interrogation frequencies [15] resulted in a strain sensitivity of 0.6 $p\varepsilon /\sqrt{Hz}$, in 5 kHz frequency bandwidth with a 10 kHz pulse repetition rate and 10 m spatial resolution. The linear chirp of the probing pulse simultaneously with laser frequency drift compensation and sinc interpolation [14,16,17,18] allowed us to achieve strain sensitivity of 3.6 $p\varepsilon /\sqrt{Hz}$ at frequencies exceeding 200 Hz and 10 kHz pulse repetition and 10 m spatial resolution.

The strain sensitivity of DAS is limited by various types of noise arising in the receiving part of the device; however, despite the progress in increasing the sensitivity, the issue of noise composition has not yet been given enough attention. Identifying the dominant noise source will allow appropriate measures to be taken to increase the DAS strain sensitivity. To date, the main sources of noise in DAS are considered to be noise caused by phase fluctuations of the laser source (usually called phase noise), as well as the shot and thermal noise of the receiving circuit. In this paper, it is shown that there is another fundamental process that leads to an increase in noise power in DAS, namely noise due to spontaneous Brillouin scattering (SpBS). Identifying the dominant noise source will allow appropriate measures to be taken to increase the DAS strain sensitivity.

The phase noise in DAS is investigated in [19]. Due to the time delay between the interfering fields backscattered by the fiber section occupied by the probing pulse, the phase fluctuations of the laser light are converted into intensity fluctuations similar to what happens in an interferometer [20]. This noise source is fundamental and is observed in all types of DAS systems [21] with direct detection [19], coherent detection [22,23], and chirped pulse [24]. Despite the fact that the phase noise can be partially compensated or averaged in DAS, the most effective way to eliminate it is to use a laser source with a high degree of coherence. It is shown that the total average noise power caused by fluctuations of the laser source contained in the DAS response is linearly proportional to the laser linewidth [19,25]. This noise source dominates whenever a relatively low-coherence laser source is used [26]. It also becomes significant even for DAS with highly coherent lasers, provided that the width of the probing pulse is sufficiently long, 100 ns or more. Additionally, phase noise increases when using a dual-pulse DAS without time delay compensation [11,19].

Other noise sources limiting the DAS strain sensitivity are determined by the design specifics. In the case of a direct detection scheme, additional noises are shot noise and thermal noise of the photodetector and preamplifier [21,27,28,29,30]. When using an erbium-doped fiber amplifier (EDFA) to increase the power of backscattered light, additional noises arise due to the effect of amplified spontaneous emission (ASE), namely ASE-shot noise, Signal-ASE beat noise, and ASE-ASE beat noise.

Here, we will focus on the conventional DAS architecture without an optical preamplifier and with direct detection, which has proven to be reliable and highly sensitive [31]. We will analyze SpBS-induced noise using a single probe pulse DAS configuration. In this scheme, a booster amplifier is used to feed as much optical power as possible into the FUT up to the threshold of nonlinear effects, in particular modulation instability (MI), which has a value of about 200 mW for long FUTs (more than 6 km), for fibers with anomalous dispersion [32], while for shorter FUTs (up to 1 km) used in our experiments, it increases to about 1 W [17].

SpBS is initiated by thermally excited spontaneous phonons in fiber [33,34,35,36,37,38]. SpBS always exists, giving rise to additional intensity noise in DAS. As it will be shown later, the SpBS-induced noise power can be comparable to the power of the phase noise. The SBS wave is even more intense and leads to a more noticeable decrease in DAS sensitivity.

The intensity of the SpBS Stokes wave grows exponentially from the initial SpBS wave over the length of the probing pulse. For the pulsed probe signal under steady-state conditions where the pulse width $T_{P}$ is much longer than the phonon lifetime $T_{B}$, $T_{P}\gg T_{B}$, the critical power when SBS begins to grow rapidly, the Brillouin threshold, can be written as [33,34]:

(1) $$P_{th}=\frac{21A_{eff}}{g_{B}L_{P}}∼7W$$

where $A_{eff}$ is the effective mode area, for Fujikura FutureGuide-LWP fiber, it is equal to $85\;{\mu m}^{2}$, $L_{P}=T_{P}v_{gr}/2$ half of the pulse length in the fiber, $v_{gr}$ is the group velocity of light in the fiber medium, $L_{p}$ = 5 m for 50 ns pulse, $g_{B}\approx 5\cdot 10^{-11}m/W$ is Brillouin gain coefficient [33,37], this value can be reduced by a factor of 2/3 if the polarization state of the pump source changes randomly due to fiber birefringence fluctuations.

## 2. Experimental Setup

**Figure 1:**
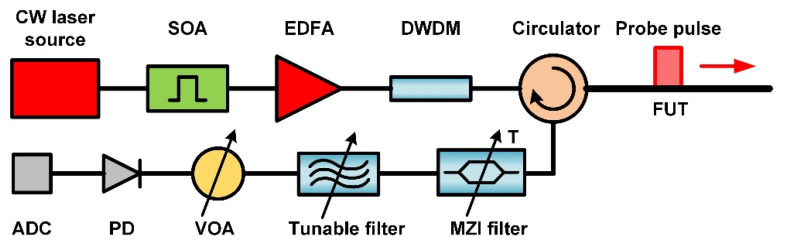
DAS experimental setup.

The noise sources in DAS were analyzed using the experimental setup shown in Figure 1.

The Koheras BASIC X15 laser module from NKT photonics (Boston, MA, USA) with 1.3 kHz linewidth was used as a coherent light source. The light was intensity modulated by a semiconductor optical amplifier (SOA) from Finisar (San Jose, CA, USA). Further pulse was amplified by an erbium-doped fiber amplifier (EDFA) and fed into a 1 km long FUT (Fujikura FutureGuide-LWP, Tokyo, Japan) through an optical dense wavelength division multiplexing (DWDM) filter with a bandwidth of 100 GHz and an optical circulator. The backscattered light that passed through the same circulator was filtered by a temperature-tunable Mach-Zehnder interferometer-based (MZI) filter with a 10 mm optical path difference corresponding to a free spectral range of about 20 GHz. Through temperature adjustment, either Rayleigh or both Brillouin waves (Stokes and anti-Stokes) can be rejected. The contrast of the MSI filter was estimated at about 27 dB. In order to select Stokes or anti-Stokes waves separately, the MZI filter was additionally supplemented with a tunable 50 GHz channel spacing filter from DiCon Fiberoptics (MTF500B-0.15-15-9-2B-N-1, Richmond, CA, USA). The filter had a bandwidth of 170 pm at the 3 dB level. After filtering, the backscattered light was further attenuated by a variable optical attenuator (VOA) and detected by a photodetector (PD) with a bandwidth of 50 MHz. The detected signal was digitized by a 12-bit analog-to-digital converter (ADC) at a sampling rate of 100 Ms/s.

## 3. SpBS Intensity

The SpBS process can be considered quantum mechanically as an interaction between an incident light photon and an acoustic phonon. In the case when a new phonon is created, the energy of the original light photon is downshifted, resulting in a photon with a reduced frequency, which is called the Stokes component of SpBS; in the case when an acoustic phonon is absorbed by a photon of the incident light, a photon with increased energy and increased frequency appears, which is called the anti-Stokes component of SpBS.

In the pure SpBS approximation, the Stokes and anti-Stokes wave intensities depend on the absolute temperature T and are described by dependencies determined by Bose–Einstein statistics [21]. However, at temperatures above several K, the intensity of Stokes and anti-Stokes waves are approximately the same. At room temperature, the difference between these intensity values is of the order of 0.1%, which is insignificant. As mentioned earlier, the SBS threshold is not reached in our OTDR setup, and the main factor limiting the intensity of the probing pulse is MI. However, the amplification of the Stokes component at the length of the probing pulse is sufficient for its intensity to exceed the intensity of the anti-Stokes component by 1–6 dB. Thus, the main contribution to the Brillouin scattering intensity is made by the Stokes component.

The theoretical consideration of the interaction between the pump wave and Stokes wave is made in [35,36,37,38]; the described model is referred to as fluctuating nonlocalized source model. The SpBS is initiated by a thermal fluctuation of the medium, which is excited by Langevin noise. The standard assumption is that this noise is described by a Gaussian random variable δ correlated in space and time with a mean of zero. Several other assumptions are also made in the model. Firstly, there is no pump wave depletion, which means that the backscattered wave has no effect on the pump wave. Secondly, the pump wave is quasi-monochromatic with narrow spectral linewidth, and it has no influence on the Brillouin gain spectrum. Thirdly, the steady-state conditions are valid, which means that the interaction time between the pump wave and the medium is much longer compared with the acoustic phonon lifetime [37]. The results of the consideration are general expressions for the spectral density of the Stokes SpBS components and their intensity at the fiber input. In the assumption of low attenuation in the fiber and low gain, the spectral density of Stokes wave is:

(2) $$S_{Stokes}\left(0,\omega \right)=G\frac{k_{B}T\nu_{S}}{A_{eff}\nu_{B}}\cdot \frac{{\left(\Gamma /2\right)}^{2}}{{\left(\omega -\omega_{B}\right)}^{2}+{\left(\Gamma /2\right)}^{2}}$$

where $G=g_{B}I_{P}\left(0\right)L=g_{B}P_{P}\left(0\right)L/A_{eff}$ is the gain factor (G≪1), $\Gamma =1/T_{B}$, $T_{B}\approx 7ns$, $I_{p}\left(0\right)$ is the pump wave intensity at the fiber input, L is the fiber length, $k_{B}$ is Boltzmann constant, T=293 K is the temperature, $\nu_{S}=\nu_{0}-\nu_{B}$ is the Stokes wave frequency, $\nu_{B}\approx 11\;\mathrm{GHz}$ is Brillouin frequency shift, $\nu_{0}=c/\lambda$. The full width at half maximum (FWHM) width of the spectrum is [32]:

(3) $$\Delta \omega_{FWHM}=\Gamma$$

The total intensity of the Stokes wave in this approximation (G≪1) is an integral of (2) over the entire spectrum [37]:

(4) $$I_{S}\left(0\right)=G\frac{kT\nu_{S}\Gamma }{4A_{eff}\nu_{B}}$$

In the case when G≫1 the total intensity of the Stokes wave is:

(5) $$I_{S}\left(0\right)=G\frac{kT\nu_{S}\Gamma }{4A_{eff}\nu_{B}}\exp\left(G/2\right)\left[I_{0}\left(G/2\right)-I_{1}\left(G/2\right)\right]$$

where $I_{j}\left(x\right)$ is the modified Bessel function of the first kind of order j.

The expressions (2) and (4) also describe the spectral density and total intensity of the SpBS Stokes wave when the pump wave is pulsed, provided that the steady-state condition is met, i.e., the pulse width is much longer than the phonon lifetime $T_{P}\gg T_{B}$. For the typical case of $T_{P}=100\;\mathrm{ns}$ $L=L_{P}=T_{P}v_{gr}/2=10\;m$ Stokes, the reflectivity can be estimated using the following:

(6) $$R_{S}=I_{S}\left(0\right)/I_{P}\left(0\right)=g_{0}L\frac{k_{B}T\nu_{S}\Gamma }{4A_{eff}\nu_{B}}\approx 1.6\cdot 10^{-8},\;\mathrm{or}\;-78\;\mathrm{dB}$$

**Figure 2:**
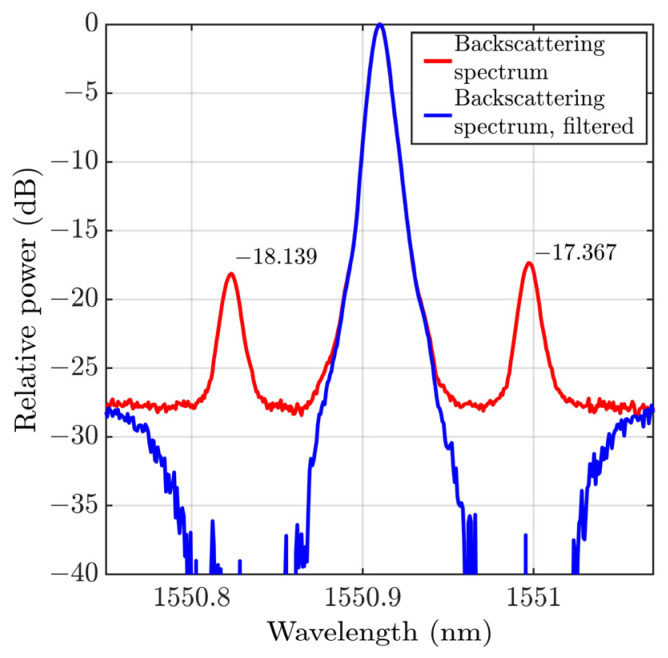
The optical power spectrum of DAS backscattered signal, without filtering (red line) and with MZI filtering (blue line). The filtering rejects the Stokes and anti-Stokes waves.

This value can be compared with the reflectivity of Rayleigh backscattering [39] with the same pulse width:

(7) $$R_{RB}=I_{RB}\left(0\right)/I_{P}\left(0\right)=\frac{1}{2}\alpha_{S}v_{gr}T_{P}S\exp\left(-\alpha_{S}v_{gr}{Τ}_{P}\right)=1\cdot 10^{-6}\;\mathrm{or}\;-60\;\mathrm{dB}$$

where $I_{RB}\left(0\right)$ is the Rayleigh backscattering intensity at the beginning of the scattering fiber segment, $\alpha_{S}=4.6052\cdot 10^{-5}\;m^{-1}$ is the attenuation constant, $S=0.25{\left(NA/n_{core}\right)}^{2}$ is the capture fraction [21], where NA≈0.14 is the numerical aperture of the fiber, $n_{core}=1.462$ and is the core refractive index. Thus, the power of the Stokes wave is about 18 dB lower than the Rayleigh backscattering power, which is confirmed experimentally. In Figure 2, the optical power spectrum of the backscattered signal is shown. As expected, the intensity of the Stokes wave is 18 dB lower than the intensity of Rayleigh backscattering. To reject the Brillouin Stokes and anti-Stokes waves, a temperature-tunable MZI filter was used; the results are also shown in Figure 2. The spectra were obtained using the ANDO AQ6319 spectrum analyzer with a resolution of 10 pm.

## 4. SpBS Intensity Fluctuations

The SpBS process is initiated by fluctuating thermal noise, and thus, the intensity of the Stokes wave also fluctuates in time [35,36,39,40]. Assuming that there is no pump depletion and low gain, the intensity fluctuations exhibit chaotic behavior with exponential statistics, which means that the Stokes field has Gaussian statistics in time.

**Figure 3:**
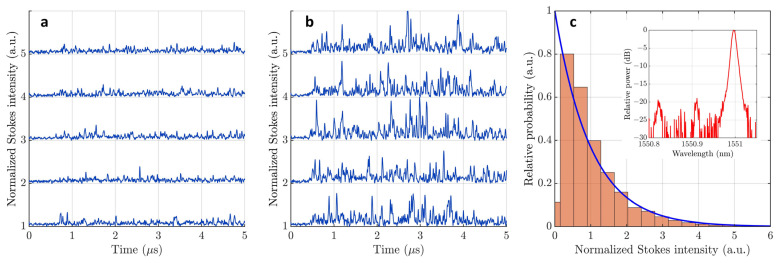
Fluctuations of the SpBS Stokes backscattering intensity at the DAS output (Brillouin OTDR trace) with low gain G<1 (**a**), fluctuations of the SpBS Stokes backscattering intensity at the DAS output with moderate gain G∼10 (**b**), a histogram that shows the statistics of SpBS intensity fluctuations (**c**), the blue line shows the exponential PDF, the inset shows the optical power spectrum of the SpBS Stokes field.

Figure 3 shows experimental realizations of Stokes intensity fluctuations along the optical fiber (Brillouin OTDR trace) at the DAS output with rejected Rayleigh and anti-Stokes backscattered fields by MZI and DICON tunable filter. The data was obtained using a 50 MHz APD photodetector and an Agilent Technologies MSO7104A oscilloscope with a sampling rate of 400 Ms/s. The DAS probing pulse width was $T_{P}=150\;\mathrm{ns}$.

Figure 3a shows five experimental realizations of SpBS Stokes intensity fluctuations in the case of low gain G<1 when the Stokes and the anti-Stokes waves have approximately equal powers (less than 2 dB difference). The Stokes signal starts at 0.7 μs time point, and it is obviously higher than photodetector noise before 0.7 μs time point.

Figure 3b shows five experimental realizations of SpBS Stokes intensity fluctuations in the case of moderate gain with (G∼10), below the SBS threshold, when the Stokes power is larger than the anti-Stokes power (6 dB difference). The histogram of the Stokes intensity fluctuations, in this case, is shown in Figure 3c. To fit the histogram, we used the exponential probability distribution function (PDF):

(8) $$p_{I}\left(I\right)=\frac{1}{\bar{I}}\exp\left(-\frac{I}{\bar{I}}\right)$$

where $\bar{I}$ is the mean intensity. As can be observed, the exponential PDF agrees well with the experimental histogram as predicted in [35,39,40]. This confirms that the SpBS Stokes field has Gaussian statistics.

The observed Brillouin OTDR trace fluctuates rapidly in time at each point along the fiber; these intensity fluctuations are incoherently added to the Rayleigh OTDR trace and can be completely considered noise.

The property of the exponential PDF is that its variance is equal to the square of the mean value. Thus, the average Stokes-induced intensity noise power is determined by the square of the mean Stokes intensity:

(9) $$P_{\begin{array}{l} Stokes\\ Intensity \end{array}}^{P}={\left(I_{S}\left(0\right)\right)}^{2}$$

The anti-Stokes wave is not correlated with the Stokes wave and has approximately the same power as Stokes in the case of low gain G≪1, as mentioned earlier. The total intensity noise power is: $P_{BS}^{P}=P_{\begin{array}{l} Stokes\\ Intensity \end{array}}^{P}+P_{\begin{array}{l} anti-Stokes\\ Intensity \end{array}}^{P}$.

This total intensity noise power due to Brillouin scattering power can be compared with other noise sources mentioned in the introduction. According to [19,25], the average intensity noise power induced by phase fluctuations of the laser source for the case of a single rectangular DAS probe pulse under the assumption of high coherency of the laser source can be estimated as:

(10) $$P_{RB}={\left(I_{RB}\left(0\right)\right)}^{2}\frac{2T_{P}}{3\tau_{coh}}$$

where $\tau_{coh}$ is the coherence time of the laser source under the assumption that the laser spectral line has a Lorentzian shape. This expression is also valid for a dual-pulse DAS [11] with compensation for the time delay between the backscattering fields. Under the assumption of a small gain, the Stokes and anti-Stokes-induced intensity noise powers are equal. Thus, the relation between SpBS-induced intensity noise and the phase noise is

(11) $$\frac{P_{RB}}{P_{BRS}}=\frac{2T_{P}}{3\tau_{coh}}\frac{1}{2}{\left(\frac{I_{RB}\left(0\right)}{I_{S}\left(0\right)}\right)}^{2}$$

It is important to note that although the ratio of Rayleigh and SpBS reflectivities is approximately equal to 18 dB (6), (7) the ratio of the width of the rectangular probe pulse and coherence time, i.e., the first ratio in the right-hand side of (11), can be low, and the two powers of noise intensity fluctuations $P_{RB}$ and $P_{BRS}$ can be comparable. In the case of a high gain (G≫1), the amplification should be taken into account and (5) should be used to estimate the Stokes intensity instead of (4) to calculate the ratio (11). Thus, in order to achieve high strain sensitivity, the elimination of Stokes and anti-stokes SpBS waves is required. The noise power increases due to amplification when the probing pulse power or width increases.

## 5. DAS Noise Experiment and Discussion

**Figure 4:**
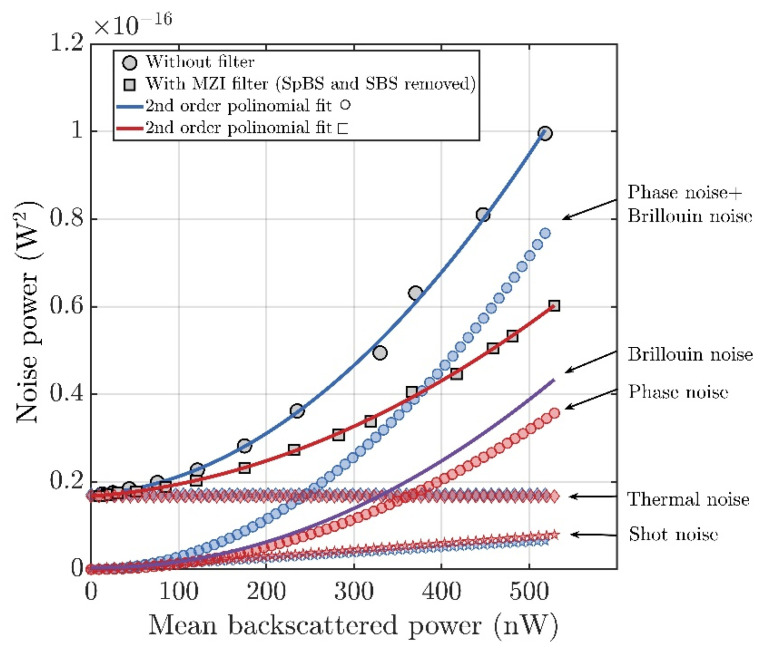
Experimental dependence of the measured optical noise power on the incident backscattered power varied using VOA without filter and with MZI filter. The continuous lines show the second-order polynomial curves which fit the experimental data. The colored diamonds, stars, and circles show the constant, linear, and square terms of the polynomial curves.

In order to experimentally compare different noise sources, we measured the averaged noise power of intensity fluctuations at the DAS output, shown in Figure 1, with a single probing pulse having the width $T_{P}=50\;\mathrm{ns}$ and fixed power. The power of the probe pulse was about 1 Watt, G∼3. A PIN photodiode with a bandwidth of 50 MHz was used as a photodetector. The optical noise power was measured depending on the backscattered power falling on the photodetector and gradually attenuated by VOA. Noise power was measured with the MZI filter, removing the Brillouin scattering components and without the filter, as shown in Figure 4. To fit the obtained data (in the sense of least squares), a second-order polynomial of the form $f\left(x\right)=ax^{2}+bx+c$ was used. This function was chosen because it describes the power dependence of the main components of total noise. The mean-square thermal noise current $j_{th}^{2}$ is independent of the intensity of the light incident on the photodetector and can be written as

(12) $$j_{th}^{2}=\frac{4k_{B}T_{R}}{R_{pd}}B_{e}$$

where $R_{pd}$ is the equivalent resistance of the photodetector, $T_{R}$ is the temperature of the equivalent resistor, $B_{e}$ is the noise equivalent bandwidth. This noise power is specified for a photodiode and, in our case, is approximately equal to $0.2\cdot 10^{-16}\;W^{2}$. The mean-square shot noise current $j_{sh}^{2}$ linearly depends on the average photocurrent $\bar{J}$:

(13) $$j_{sh}^{2}=2e\bar{J}B_{pd}$$

where e is the electron charge, $B_{pd}$ is the bandwidth of the photodetector. Note that (13) is valid only for PIN photodiode in the approximation of zero dark current. The photocurrent J is proportional to the optical power falling on the photodetector $P_{opt}$:

(14) $$J=\frac{\eta e}{h\nu }P_{opt}$$

where h is Planck constant, ν is optical frequency, η is the quantum efficiency of the photodetector. The noise powers due to SpBS (9) and phase noise (10) are proportional to the square of the Rayleigh backscattering intensity.

Taking into account the above considerations, the terms of the polynomial with coefficients a, b, and c can be interpreted as follows: the term c is a component of the noise power that does not depend on the incident backscattered power; this term can be attributed to the thermal noise power of the receiving circuit; the term bx linearly depends on the incident backscattered power this term can be attributed to the shot-noise power; the term $ax^{2}$ has the square dependence on the incident backscattered power and can be attributed to the noise power due to laser phase fluctuations (phase noise) and Brillouin intensity fluctuations.

Figure 4 shows the noise components measured with and without an MZI filter. The units of measurement of optical noise power are [W^2^] since the units of measurement of the fluctuating value are optical watts [W]. It can be seen that thermal noise components shown by diamonds (blue and red) coincide in both cases. The same is true for the shot noise components shown by asterisks (blue and red). The square components shown by circles (blue and red) are different because the blue curve includes noise caused by Brillouin scattering, while the red curve does not. An estimate of the noise caused by Brillouin scattering can be obtained by subtracting the red curve from the blue curve; the result is shown in Figure 4 of the purple curve. As can be observed in this particular case, the noise due to SpBS (signed as Brillouin noise) is about 23% higher than the noise due to laser phase fluctuations (signed as Phase noise). The absolute values of the SpBS-induced noise are lower than theoretically estimated. It can be explained by the relatively low ADC sampling rate of 100 Ms/s. Insufficient sampling rate leads to incorrect estimation of the amplitude of the broadband signals. Additionally, the non-flat spectral characteristic of the photodetector and a decrease in the frequency response at a high frequency can lead to a decrease in the reception of the Brillouin signal.

Thus, Brillouin scattering rejection in front of the photodetector helps to reduce DAS noise. This statement is most relevant in the case of using short probing pulses (to obtain the minimum value of the gauge length), where a broadband PD with a bandwidth of the order of 30 MHz is required.

Based on the data obtained, it is possible to estimate the expected increase in strain sensitivity using the results presented in [26]. The strain sensitivity is estimated under the condition that the average power of the useful signal is equal to the average noise power. Therefore, the increase in strain sensitivity corresponds to the square root of the decrease in noise power. In our case, when the noise caused by Brillouin scattering is rejected, the total noise power decreases by 1.7 times, and the sensitivity increases by 1.3 times.

In addition to spectral filtering, the relative effect of Brillouin scattering-induced noise can be reduced by using single-mode optical fiber with ultra-weak fiber Bragg gratings [41]. With a typical reflectivity value of −30 dB for each FBG, the useful reflected signal that replaces the Rayleigh backscattering signal is 30 dB (in the case of 100 ns probe pulse width) higher than the Rayleigh scattering signal; therefore, 58 dB higher than the Brillouin scattering signal. In this case, the Brillouin-scattering-induced noise can be neglected. Unfortunately, this option is not universal, especially in the case of pre-installed fiber.

## 6. Conclusions

The DAS noise composition has been experimentally studied for a specific case of a short (1000 m) FUT. This case is typical for engineering geology. It is shown that along with the known noise sources, namely thermal, shot, and phase noise, Brillouin backscattering-induced noise makes a significant contribution. The noise level is theoretically estimated. It is shown experimentally that the intensity of the Brillouin waves fluctuates, and the probability distribution of these fluctuations is described by a negative exponential PDF. The resulting average noise power of these fluctuations is equal to the square of the average Brillouin intensity, which is about 18 dB lower than the Rayleigh backscattering power of Stokes and anti-Stokes waves. In our case, the noise from Brillouin scattering is dominant. The reduction of the overall noise level by rejecting the Brillouin components of the scattered field using an asymmetric MZI is demonstrated. Such filtering is relevant for small gauge lengths and would increase the DAS strain sensitivity.

## Data Availability

The data presented in this study are available on request from the corresponding author.
